# Bisphenol A (BPA) Leading to Obesity and Cardiovascular Complications: A Compilation of Current In Vivo Study

**DOI:** 10.3390/ijms23062969

**Published:** 2022-03-09

**Authors:** Ruth Naomi, Muhammad Dain Yazid, Hasnah Bahari, Yong Yoke Keong, Retnagowri Rajandram, Hashim Embong, Soo Huat Teoh, Shariff Halim, Fezah Othman

**Affiliations:** 1 Department of Human Anatomy, Faculty of Medicine and Health Sciences, Universiti Putra Malaysia, Serdang 43400, Malaysia; ruthmanuel2104@gmail.com (R.N.); haba@upm.edu.my (H.B.); yoke_keong@upm.edu.my (Y.Y.K.); 2 Centre for Tissue Engineering and Regenerative Medicine, Faculty of Medicine, Universiti Kebangsaan Malaysia, Cheras, Kuala Lumpur 56000, Malaysia; dain@ukm.edu.my; 3 Department of Surgery, Faculty of Medicine, Universiti Malaya, Kuala Lumpur 50603, Malaysia; retnagowri@ummc.edu.my; 4 Department of Emergency Medicine, Faculty of Medicine, Universiti Kebangsaan Malaysia, Kuala Lumpur 56000, Malaysia; hashimembong77@ukm.edu.my; 5 Advanced Medical and Dental Institute, Universiti Sains Malaysia, Penang 13200, Malaysia; soohuat@usm.my; 6 Neuroscience Research Group, International Medical School, Management & Science University, University Drive, Off Persiaran Olahraga, Shah Alam 40100, Malaysia; drhalim_shariff@msu.edu.my; 7 Department of Biomedical Sciences, Faculty of Medicine and Health Sciences, Universiti Putra Malaysia (UPM), Serdang 43400, Malaysia

**Keywords:** endocrine disruptors, obesity, neuroimmune signals, cardiovascular complications, transgenerational effects, biomarkers

## Abstract

BPA is one of the most common endocrine disruptors that is widely being manufactured daily nationwide. Although scientific evidence supports claims of negative effects of BPA on humans, there is also evidence suggesting that a low level of BPA is safe. However, numerous in vivo trials contraindicate with this claim and there is a high possibility of BPA exposure could lead to obesity. It has been speculated that this does not stop with the exposed subjects only, but may also cause transgenerational effects. Direct disruption of endocrine regulation, neuroimmune and signaling pathways, as well as gut microbiata, has been identified to be interrupted by BPA exposure, leading to overweight or obesity. In these instances, cardiovascular complications are one of the primary notable clinical signs. In regard to this claim, this review paper discusses the role of BPA on obesity in the perspective of endocrine disruptions and possible cardiovascular complications that may arise due to BPA. Thus, the aim of this review is to outline the changes in gut microbiota and neuroimmune or signaling mechanisms involved in obesity in relation to BPA. To identify potentially relevant articles, a depth search was done on the databases Nature, PubMed, Wiley Online Library, and Medline & Ovid from the past 5 years. According to Boolean operator guideline, selected keywords such as (1) BPA OR environmental chemical AND fat OR LDL OR obese AND transgenerational effects or phenocopy (2) Endocrine disruptors OR chemical AND lipodystrophy AND phenocopy (3) Lipid profile OR weight changes AND cardiovascular effect (4) BPA AND neuroimmune OR gene signaling, were used as search terms. Upon screening, 11 articles were finalized to be further reviewed and data extraction tables containing information on (1) the type of animal model (2) duration and dosage of BPA exposure (3) changes in the lipid profile or weight (4) genes, signaling mechanism, or any neuroimmune signal involved, and (5) transgenerational effects were created. In toto, the study indicates there are high chances of BPA exposure affecting lipid profile and gene associated with lipolysis, leading to obesity. Therefore, this scoping review recapitulates the possible effects of BPA that may lead to obesity with the evidence of current in vivo trials. The biomarkers, safety concerns, recommended dosage, and the impact of COVID-19 on BPA are also briefly described.

## 1. Introduction

Bisphenol A (BPA), also known as an obesogen, is a commonly used industrial chemical for plastic-based production. Despite the fact that the Food and Drug Administration (FDA) claims BPA is safe for consumers in an acceptable range (50 mcg/kg) in manufacturing, it still raises concerns among researchers [1]. Although it has been used for decades, recent discovery shows that even a small quantity of BPA exposure can interfere with normal homeostasis in the body over time. Particularly, numerous prospective clinical data and in vivo trials have proven that BPA could lead to endocrine disruptions, thereby promoting lipid accumulations, causing obesity as a commonly notable sign. The negative effect not only stops with the exposed subjects but also results in transgenerational effects. Studies claim that up to 85% of those BPA product consumers tend to be obese and at least 59% of those subjects tend to develop large waist circumference [2,3,4]. A recent study shows that cardiovascular complications such as angina, hypertension, heart attack, and peripheral artery disease are common among subjects exposed to BPA for a long time [5]. Even drinking water in a polycarbonate bottle just for two weeks can raise the BPA in urine to up to two-thirds [6]. In consideration of this claim, in the year 2010, the National Toxicology unit classified BPA as a toxic chemical while FDA went along with the decision in 2018 and declared that they did not find any evidence suggesting that BPA was safe for consumption [7]. Correspondingly, the World Health Organization (WHO) points out the negative outcome of BPA consumption and motivates deep investigations on BPA.

### Bisphenol A

BPA (2,2-bis(4-hydroxyphenyl)propane) is a chemical that is produced from the condensation of acetone and phenol. It comprises two hydroxyphenyl groups, which are derived from bisphenols and grouped as diphenylmethane. It is insoluble in water and highly soluble in organic solvents. Exposure to extreme heat eases the release of BPA. It is the most commonly used element, particularly for plastic-based manufacturing [8]. According to the global BPA market report in 2018, BPA annual production increased by 3% and it is expected that it could reach up to 7348 kg tonnes by the year 2023. Of these, North East Asia is the highest in the list of countries that consume the most BPA [9]. This may also serve as one of the primary reasons for Asia countries to be among the fattest nations in comparison with other countries globally. BPA is also known as xenoestrogen due to its ability to imitate estrogens in the human body. This is most likely because of the similarity of phenol groups which are present in BPA and estrogen. This likeness makes the synthetic compound interfere and stimulate the estrogenic pathways. This is achieved by the binding of BPA to estrogen receptors (ER) such as ERα, and ERβ. Despite being a selective modulator for estrogen receptors, an excessive level of BPA can cause it to freely bind with androgen receptors as well [8].

Usually, subjects tend to be exposed to BPA through food, like leaching from water bottles or plastic food containers. This could be due to the slow decomposing of polymer bonds in the polycarbonates which allows the release of unpolymerized monomers to be mixed with liquids or foods. This toxicant has a very poor understanding of its toxicokinetics mechanism which makes the complete elimination of the BPA impossible from the human body [10]. However, BPA can be eliminated via urine upon conjugation in the liver within 24 h, after a half-life of at least 5.3 h. In most cases, BPA tends to accumulate in different tissues such as the placenta, lungs, kidney, and liver [11]. In these respective tissues, β-glucuronidase enzyme exists in a very high concentration. As such, this enzyme will deconjugate BPA, and make it to exist freely in its active form. Since BPA naturally possesses lipophilic affinity, it will bind to fats in a high affinity, further leading to its bioaccumulation [10] and promoting adipogenesis [11].

## 2. Methods

### 2.1. Search Strategy

The search was done based on the protocol described in Preferred Reporting Items for Systematic Reviews and Meta-Analyses (PRISMA) [12]. The search was done systematically to identify potentially relevant journals associated with BPA and obesity from the view of endocrine disruptions. For these, a few databases were selected such as Wiley Online Library, Nature, Medline & Ovid, and PubMed. Google Scholar was used as the platform to cross-check our articles to not leave out any potentially relevant articles. The article search was limited to the past five years. Boolean operator guidelines were followed to select and perform the keywords search [13]. These are as described as: (1) BPA OR environmental chemical AND fat OR LDL OR obese AND transgenerational effects or phenocopy (2) Endocrine disruptors OR chemical AND lipodystrophy AND phenocopy (3) Lipid profile OR weight changes AND cardiovascular effect (4) BPA AND neuroimmune OR gene signaling.

### 2.2. Inclusion Criteria

Only original articles discussing in vivo studies were included in this review. All selected articles were written in English with the presence of an abstract. The articles selection was limited from January 2017 till 15 January 2022, to give recent evidence on the selected topic. The selected articles must precisely provide all the findings. This includes (1) the type of animal model (2) duration and dosage of BPA exposure (3) changes in the lipid profile or weight or cardiovascular changes (4) genes, signaling mechanism, or any neuroimmune signal involved, and (5) transgenerational effects.

### 2.3. Exclusion Criteria

All review articles, theses, conference proceedings, patents, and articles written in any other language apart from English were excluded from being further reviewed. Those articles that did not meet the inclusion criteria were also excluded. Any study focusing on retrospective data with an unclear dosage or duration of BPA exposure was also excluded; this was the main reason for clinical data being excluded. Data extraction was done independently by five authors (R.N, H.B, M.D.Y, H.E, and R.H). All extracted data were compiled together and any arise disagreements were further discussed with other authors (R.R, S.H.T, and S.H) until mutual understanding was achieved. The finalized data extraction was tabulated as shown in Table 1. It contained information on (1) type of animal model (2) type of BPA exposure (3) duration of exposure and dosage of exposure, and (4) the summary of the findings. No conflict of interest was found among the authors during the data extraction.

## 3. Results

### 3.1. Literature Search

**Figure 1:**
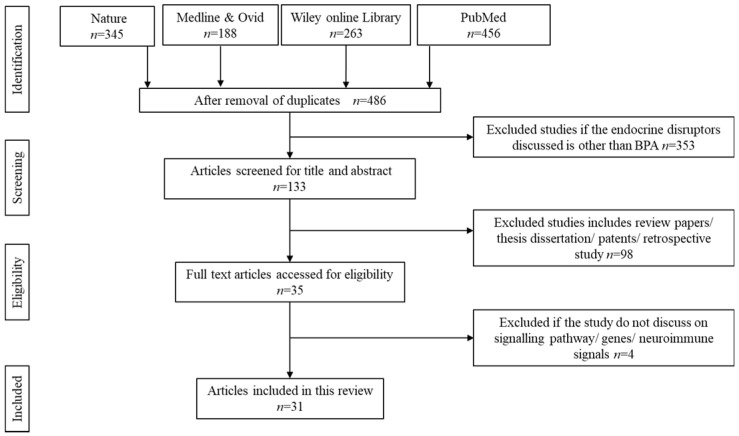
Identification and screening for literature search.

The initial search resulted in 1252 potentially relevant articles. Upon thorough screening, 766 articles were removed owing to duplication and unmatched search contents. Another 353 articles were further removed upon screening the title and abstracts. Only titles containing BPA as the endocrine disruptors were considered to be retained. However, another 98 search results were eliminated upon confirmation as review papers, theses, dissertations, patents, copyrights, and articles discussing retrospective data based on clinical writings. Upon deep screening, 31 were finalized to be reviewed in detail. Figure 1 summarizes the screening for the literature search.

### 3.2. BPA and Obesity

Obesity is one of the emerging health conditions worldwide, specifically in Asia. Obesity is simply defined as the abnormal accumulation of fat. However, considering body mass index, this may differ between different groups of population based on their height and weight. While lack of exercise and poor nutritional intake could be some reasons for obesity, one of the external reasons could be due to endocrine disruptions [25]. Past scientific studies have proven that endocrine disruptions could lead to obesity. This endocrine disruption mechanism in most conditions results in transgenerational effects. BPA increases the risk of obesity via altering the normal pathway of endocrine (metabolic pathway) in the adipose tissue.

BPA, an estrogenic chemical, is able to hinder the release of adiponectin by binding with estrogen receptors specifically with ERα and ERβ. By acting on adipocytes and infiltrating macrophages, BPA lowers adiponectin and elevates inflammatory cytokines [26]. In this context, the presence of inflammatory cytokines, such as IL-6 and TNFα, promote inflammation in the adipose tissue, hindering lipolysis. In this case, the lipid overflow towards the oxidative tissues like skeletal muscles and liver will promote ectopic fat distribution, thereby leading to abdominal obesity [27]. Besides, certain genes responsible for lipid metabolism are downregulated in the case of BPA binding to estrogen receptors. This includes fatty acid binding protein 4 (*FABP4*) and a cluster of differentiation 36 (*CD36)* [28]. *FABP4,* known as a novel adipokine, is a cytoplasmic protein produced by the mature adipocytes that have the ability to bind to hydrophobic ligands [29] such as in lipid A [30]. Lipid A is an endotoxin and it is known for its ability to anchor to the hydrophobic surface of lipopolysaccharide [31]. By binding with lipid A, *FABP4* enhances the accumulation of fatty acids and causes high concentration gradients with the cell membranes. This fatty acid deposition will eventually combine with the unsaturated parts of the long chain fatty acids, leading to even distribution of lipids to other organelles, [32], further preventing abnormal fat deposition. Upregulation of *FABP4* in adipose tissue may reduce the transportation of free fatty acids. This prevents the breakdown of free fatty acids or β oxidation. As a result, since there is no breakdown of free fatty acids, this may accumulate inside the adipose tissue, exceeding its normal capacity. Eventually, this will overspill to other organs such as the liver. In the liver, the normal function facilitating the storage of triglycerides is interrupted, leading to toxic reactive species formation due to excessive level of lipids. In due course, lipotoxicity emerges [33].

Aside from this, *FABP4* is closely associated with a gene known as proprotein convertase subtilisin/kexin type 1 (PCSK1), that encodes proprotein convertase PC1/3. Downregulation of *FABP4* will result in the reduction of PCSK1, thereby leading to the deficiency of PC1/3 [28]. Since the primary role of PC1/3 is in the initial step of insulin biosynthesis, the deficiency of PC1/3 could impair the proteolytic cleavage of proinsulin [34]. Excessive levels of proinsulin will stimulate hyperphagia, causing expansion of fat cells mass and accumulation of lipids. This is because insulin possesses the ability to inhibit lipolysis [35]. Conversely, *CD36* is an important regulator for *CD36* is one of the long chain fatty acids in the plasma membrane of the adipose tissue. As such, over expression of *CD36* can cause flux to the long chain fatty acids, leading to visceral obesity [36].

### 3.3. BPA, Obesity and Neuroimmune Signals

**Figure 2:**
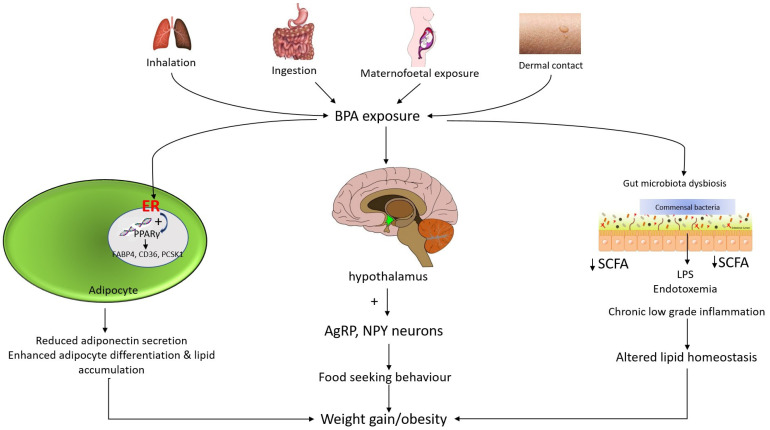
Mechanism of action of bisphenol A and associated obesity. Mechanism of BPA-induced weight gain may be due to its estrogenic activity. BPA binds to estrogen receptors (ERα and ERβ) and subsequently induces a perturbation in peroxisome proliferator-activated receptor gamma (PPARγ) signaling. BPA increases the number and size of adipocytes by regulating the expression of genes such as fatty acid binding protein 4 (FABP4), cluster of differentiation 36 (CD36) and proprotein convertase subtilisin/kexin type 1 (PCSK1). In addition, the estrogenic effect of BPA inhibits adiponectin secretion. Meanwhile, in utero and adult exposure to BPA affect the hypothalamic Agouti-related peptide (AgRP) and neuropeptide Y (NPY) neurons. These potent neuropeptides have a stimulating effect on the appetite. BPA that accumulates in the gut may contribute to gut bacterial dysbiosis. BPA exposure reduces gut small chain fatty acid (SCFA) and increases systemic lipopolysaccharide (LPS) levels, leading to chronic low-grade inflammation and subsequently altered lipid homeostasis.

BPA ingestions result in endocrine disruptions and cause an interruption in neuroimmune signals in the central nervous system (CNS), in association with obesity. Exposure to BPA could affect the hypothalamic circuitry in CNS which is proven to stimulate the expression of Agouti related peptide (AgRP) and neuropeptide Y (NPY). In contrast, the level of proopiomelanocortin (POMC) will be reduced [37]. AgRP is an orexigenic peptide that becomes over-activated in the arcuate nucleus of the hypothalamus. AgRP neurons comprise a few downstream neuronal pathways which become suppressed by the AgRP neuron activation. The downstream neuronal signals are the appetite regulator or suppressor. For instance, an activated AgRP neuron will block the action of anorexigenic neurons (appetite suppressing neurons) at the nucleus of the paraventricular thalamus. Thereby, a hunger-like response pattern is triggered in the insular cortex [38]. In contrast, AgRP neurons have been found to control appetite by hindering the action of anorexigenic neurons in the parabrachial nucleus (PBN) that produces calcitonin gene-related peptide (CGRP) by the photostimulation formed by AgRP axonal fibers. Parabrachial CGRP is responsible for controlling satiety. Inhibition of CGRP neurons may increase food intake and frequency while decreasing sensitivity to the anorexic effects of meal-related satiety peptides in a compensatory way [39]. Apart from that, AgRP neurons induce hunger via projecting to the downstream signaling pathway namely in the lateral hypothalamus, bed nucleus of the stria terminalis, and thalamic and hypothalamic nucleus in the paraventricular [38]. Figure 2 illustrates the potential molecular and mechanistic links between BPA and obesity.

BPA can cause the arcuate nucleus’ cell bodies to generate excessive levels of NPY, which is subsequently transmitted and deposited into the paraventricular nucleus. There, it stimulates the outflow of efferent impulses in the paraventricular nucleus, resulting in an increase in hunger [40]. In these, Y1 receptors, also known as lipid modulators, are responsible for NPY’s appetite-stimulating effect [41]. NPY can also trigger lipogenic enzymes in the adipose tissues, which contributes to obesity development. As a result, NPY stimulates and controls the formation of adipose tissue in a direct manner. Pre-adipocyte proliferation has been linked to the excessive expression of the local NPY in the visceral. Thus, through the stimulation of the Y1 receptor, this adipose tissue tends to grow in size [42]. Furthermore, triggering of the Y2 receptor in the NPY might result in the formation and accumulation of adipose tissue. The Y2 receptor ligand, also known as gut-derived hormone peptide YY (PYY), is produced postprandially from L cells into the distal intestine [42].

### 3.4. Signaling Pathway of BPA in Obesity

Studies have proven that BPA could stimulate genes associated with lipid metabolism in humans via the deactivation of the peroxisome proliferator-activated receptors alpha (PPARα) signaling pathway. This may further trigger the expression of *CD36*, and *FABP4* in the adipose tissue. PPARα is an important element in the catabolism of fatty acids and this process occurs via the expression of genes related to oxidation of the mitochondrial and peroxisomal fatty acids. Conversely, when PPARα is suppressed, lipids tend to accumulate in the hepatocytes, further aggravating obesity. Alternatively, BPA could prompt adipocytes dysfunction by acting directly on PPARγ. Generally, PPARγ is known for its prime modulation activity on adipogenesis, specifically isoform PPARγ2 which is present abundantly in adipose tissue. Upon stimulation, PPARγ will further activate its downstream signaling molecules such as phosphoenolpyruvate carboxykinase, lipoprotein lipase, CD36, and aquaporin 7, leading to excessive adipocyte differentiation, thereby interfering in the lipid metabolism process [43]. Studies show that phosphoenolpyruvate carboxykinase promoter activation could lead to the development of lipodystrophy, due to the extreme level of lipid droplets in the brown adipose tissue [44] while the high level of lipoprotein lipase may induce assimilation of triglycerides [45].

Besides, BPA disrupts the metabolism of lipid by increasing sterol regulatory element-binding protein 1 (SREBP1). SREBP1 is an essential transcription factor that regulates fatty acid metabolism via the modulation of genes responsible for lipolysis and lipogenesis such as *FAS* and *SCD*. Primarily, SREBP1 exists in the inactive form binding to the endoplasmic reticulum together with SREBP cleavage activating protein (SCAP), forming a coatamer protein II vesicles. This will then enter the Golgi apparatus to become the mature form of n-SREBP-1 from p-SREBP-1. The matured form will then enter the nucleus and actively bind with sterol regulatory elements and activate targeted genes [46]. In the case of increased levels of sterol in the cellular region, SREBP is hindered from entering the nucleus. Thus, the targeted genes are not activated, leading to accumulations of lipids in the liver [47]. Anyhow, BPA stimulates the adipogenic signaling pathway by inducing the overexpression of CEBPA. CEBPA is known for its role in adipose tissue differentiation and its overexpression will eventually stimulate its downstream pathway involving hexokinase 2, CD36, and lipoprotein lipase [48].

In fact, lipid profile is greatly altered with BPA exposure. Experiments by Santangeli et al. (2018) prove that zebrafish exposed to BPA show decreased level of DGAT2 [18]. DGAT2 gene is responsible for β oxidation and synthesis of triglycerides, and changes in the DGAT2 level inversely correlate with the changes in the size of the fat cells [49]. Comparatively, Pu et al. (2017) witnessed sex-specific gene alteration in subjects exposed to BPA. They discovered that BPA could extensively upregulate estrogen receptor 1 (*ESR1*) and glucocorticoid receptor (*GR*) genes in female preadipocytes compared to the male preadipocytes. In this case, *ESR1* is also known as a regulator of vascular endothelial growth factor A (VEGFA). VEGFA functions predominantly in the regulation of angiogenesis of adipose tissue. As such, over expression of *ESR1* and *GR* increase adipogenic differentiation [20]. This claim was further proved by Caporossi et al. (2015) who witnessed a similar outcome, which may be quoted as females being more susceptible to health effects such as obesity compared to males due to BPA exposure [50]. Furthermore, *CB1* could also be one of the targets of BPA in association with obesity development. It has been hypothesized that *CB1* upregulation could stimulate the appetite, cause lipids to stock up together, and inhibit lipolysis by compromising the action of PPARα.

Along with other studies, Tian et al. (2021) noticed a decreased level of G-protein coupled receptor 55 (*gpr55*) and fatty acid amide hydrolase in zebrafish subjected to BPA in the static water system [51]. The *gpr55* gene may bind to *CB1* and lysophosphatidylinositol, an endogenous ligand, with high affinity in order to activate its downstream signaling pathway. The expression of lipogenic genes in visceral and subcutaneous adipose tissue will be stimulated as a result of this binding [52]. Meanwhile, decreased level of fatty acid amide hydrolase was able to enhance the system signaling of endocannabinoids, thus triggering the frequency of hunger [53]. Apart from this, the decrease of zinc α 2 glycoprotein (ZAG), hormone-sensitive lipase (HSL), and elevation of TLR4 and NF-κB are some other indications of BPA interference with lipid profile [24]. ZAG protein plays an important role in the mobilization of adipose tissue, thereby modulating lipid metabolism to maintain body weight. Reduced ZAG levels, on the other hand, are linked to lipogenesis activation and lipolysis inhibition, as well as hepatic fat storage and dyslipidemia [24].

Into the bargain, BPA induced reduction of HSL, causing the lipolysis to be inhibited as well. HSL’s major function is to facilitate the hydrolysis of diacylglycerol and triacylglycerol. HSL is usually phosphorylated during the hydrolysis process by extracellular-regulated kinase and protein kinase A. Thus, the lipolytic enzyme is activated, which improves the conversion of HSLs into lipid droplets. In this context, more of its hydrophobic surface is exposed, making lipid droplet adhesion to the substrate easier. In short, this mechanism promotes lipolysis. Disruption or a reduction in HSL levels, on the other hand, interferes with the metabolic phenotypes and slows down lipolysis [54]. Increased level of TLR4 and NF-κB is another signaling pathway manifested in obesity induction due to BPA exposure [24]. TLR4 elevation has been shown to stimulate downstream pathway proteins such as IKK-NF-B and JNK via the MyD88 and non-MyD88 routes. Some agonists, including lipopeptides, lipopolysaccharides, saturated fatty acids, and oxidised low-density lipoprotein, eventually bind to TLR and cause adipose tissue to expand. Such expansion of adipose tissue causes the monocyte chemotactic protein-1, inflammatory factors, and free fatty acids to be released, resulting in obesity [55].

### 3.5. BPA and Dysregulation of Gut Microbiome

BPA has been shown to cause intestinal dysbiosis in obese people through interference with estrogen receptors. Microbiome acts as substrates for xenobiotics, microbial metabolites, and also for estrogen molecules. In this instance, estrogen does play an important role in bacterial colonization in the gut. This is because, estrogen can stimulate the formation of mucous in the intestine which may further allow beneficial bacteria to populate in the gut, thereby enhancing the integrity of the gut lumen [51]. Alternatively, estrogen has the potential to selectively allow the growth of bacteria in the gut [56], and this effect is commonly observable during BPA exposure. BPA exposure during pregnancy, in particular, promotes the evolution of obesity phenotypes in rats by disrupting the gut flora. Investigations in SD rats reveal that changes in normal gut flora promote lipogenesis by compromising gut–brain integrity, as reported in Table 1. The lower amount of IgA and development of pIgR support this theory [15,19]. IgA regulates gut homeostasis by maintaining the permeability of the gut barrier, which is critical for gut homeostasis. In reality, the abundance of beneficial bacteria in the gut has an impact on the establishment of the gut barrier [19]. *Bifdobacterium* spp. are vital for reducing total cholesterol [57], pro-inflammatory cytokines in adipose tissues, and maintaining a regular lipid metabolism process [58]. Obese subjects exposed to BPA had lower levels of *Bifdobacterium* spp [59], *Firmicutes* phylum, specifically *Clostridium butyricum, Clostridium Cluster XIV, Clostridium butyricum*, and *Clostridium Cluster XIVa* [19]. Butyrate, a key component in the expression of the leptin gene, is produced by *Firmicutes* [60]. As a result, a lower amount of *Firmicutes* lowers the leptin levels and raises appetites. *Clostridium* spp., on the other hand, regulates lipid metabolism, inhibits lipid buildup in the liver, and reduces the formation of short-chain fatty acids [61]. Because BPA reduces *Clostridium* spp, it has the inverse effect in the gut. Particularly, the level of *Firmicutes* often is related to leucocytes DNA methylation in the genes responsible for lipid metabolism and obesity in pregnant mothers [62].

On the flip side, sex-dependent BPA exposure results in differences in gut microbial composition. Male offspring, for example, have an abundance of *Bacteroides*, *Mollicutes*, *Prevotellaceae*, *Erysipelotrichaceae*, *Akkermansia*, *Methanobrevibacter*, and *Sutterella*, whereas female offspring have an abundance of *Lachnobacterium* spp. and *Prevotella* spp. Both genders’ perinatal were exposed to the same dose (50 mg/kg) and the same period of BPA in this study [63]. This implies that BPA causes sex-dependent microbial dysregulation. This assertion is backed up by research conducted by Wu et al. in 2020, who observe the similar effects of BPA on male and female obesity phenotypic development [64]. Succinctly, the pro-inflammatory gut microbiome is commonly observed in female subjects while the anti-inflammatory microbiome is seen in male subjects exposed to BPA [59]. Conversely, In contrast, both genders of progeny have higher levels of CKC4 protein, which increases triglyceride accumulation in the muscle [65]. Another study found that BPA promotes *Proteobacteria* colonization and reduction of phylum Tenericutes [59]. *Proteobacteria* overgrowth indicates gut dysbiosis, which might indicate increased inflammation [66] and permeability in the colon [67] as well as increased fat storage [68].

Simultaneously, prenatal BPA exposure raises lipopolysaccharide levels and lowers the variety of gut bacteria and metabolites such as short chain fatty acids in progeny. Reddivari et al. (2017) observe that BPA exposure of 200 µg/kg/day triggers inflammation in the liver and intestine and increased the systemic lipopolysaccharides via gut microbiome dysregulation. An elevated level of *Methanobrevibacter* is seen in prenatal exposure to BPA, which is closely associated with a high level of energy intake and weight gain. To add, some of the short chain fatty acid-producing bacteria such as *Ruminococcaceae*, *Odoribacter* spp., and *Oscillospira* spp. are drastically reduced in BPA-exposed subjects [69]. Short chain fatty acids play a vital role in lipid homeostatic and prevent diet-induced obesity via the releasing of anorectic hormones and by raising the energy expenditure [70]. Adult *Canis familiaris* exposed with BPA for 2 weeks exhibited a high level of *Bacteroides ovatus, Ruminococcus* spp., and *Cetobacterium somerae* along with a reduction in beneficial microbes such as *Flexispira* spp and *Bacteroides* spp. This normal shift of bacteria in the gut may lead to leaky gut, causing harmful microbes metabolites to cross the blood–brain barrier, further enhancing the development of disease [62].

### 3.6. BPA and Transgenerational Effects of Obesity

**Figure 3:**
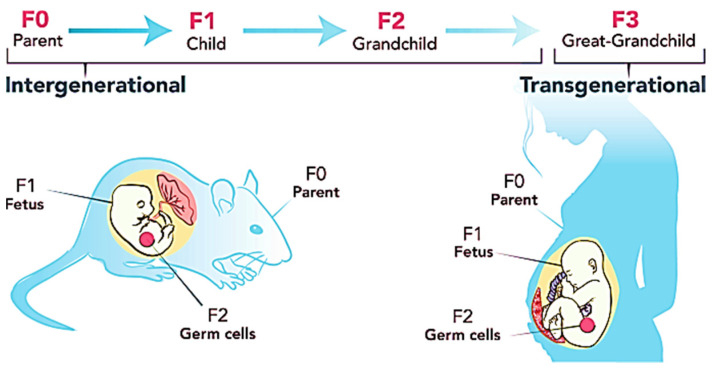
Transgenerational inheritance pattern of obesity. Figure reused under the permission granted by http://creativecommons.org/licenses/by/4.0/ (accessed on 21 February 2022) [82].

BPA, a reprotoxicant, increases the risk of sperm and ova cell malformation in those who are exposed to it. Tail impairment, sperm hook curvature, poor sperm morphology, follicle cell loss in the primordial, and cell loss in the stroma are just a few examples of data supporting this claim. This type of anomaly can be handed on through the F1 lineages [16]. This is because BPA has the ability to induce obesity to be passed down through the generations through epigenetic transgenerational inheritance. As indicated in Table 1, all researchers agree that BPA exposure may be passed down from the male and female germlines to the progeny. The epiphenotype’s effects can be carried down up to F5 generations [71]. Figure 3 shows the inheritance pattern of obesity from F0 generation up to the offspring. The transgenerational effects occur mainly due to DNA methylation of the CpG dinucleotides [72] which is closely associated with genomic imprinting [73]. This imprinting is permanently programmed and unable to be reversed or reprogrammed after fertilization which eases the transmission of defected genes to the offspring [74]. Those genes can be either inherited from the maternal or paternal side. Exposure to 50 μg/kg/day in the long term has been proven to decrease lysine and histone acetylation of H3K9, H3K27, and H4K12. Conversely, the level of deacetylase Sirt1 is increased, which in turn enhances the binding of ERβ to caveolin 1. This is one of the mechanisms of epigenetic inheritance in the offspring due to BPA exposure [73].

BPA may change and rearrange transcription factors in the germline during embryo development, resulting in the demethylation of DNA in the genome. Demethylated DNA permits transcription factors to connect to it readily, which should not happen. This binding will eventually prevent DNA from being remethylated, resulting in the alterations being maintained in the adult germline. The CTCF motif is found in area 107 in sperm, which is where this activity normally takes place [75]. This is due to the presence of progenitors and mature adipocytes in the CTCF locus [71]. This defect might then be passed on to the embryo during fertilisation, resulting in the persistence of transcriptional alterations during the embryogenesis stage. Upregulation of genes that cause deformity, notably in cell physiology, liver morphology, and fat tissue morphology, will eventually be passed on to the progeny. Epigenetic transgenerational inheritance of the changed phenotypes is partly attributable to germline-mediated epimutations [76]. Glial cell-derived neurotrophic factor (*Gdnf*) and estrogen-related receptor alpha (*Esrra*) are the most typically differentially methylation genes linked to adult onset obesity [74].

Conversely, if identical transcription factor alterations are present in the oocytes, the obesity epiphenotype can be handed down through the maternal germline. If both the male and female are exposed to BPA, a faulty transcription factor is found in both the sperm and the oocytes, making it easier for the transgenerational impact to be passed on to the progeny. In such instances, FTO sites will be triggered, and its binding sites will be exposed, allowing BPA to bind to these receptors in the chromatin area of primordial germ cells. This generally happens after the DNA has previously been demethylated. As a result, DNA remethylation is inhibited, throughout the gestation period or after E13.5 when the germline is developing [71]. Furthermore, some research suggests that BPA-dependent FTO enhancers are located in the ARC area of the hypothalamus, which is implicated in hunger regulation [71]. Another possible explanation for obesity’s transgenerational impact might be BPA’s activation of the intronic enhancer of FTO. As a result, the amount of m^6^A is lowered, and this chromatin-associated eRNA will persist until the intergenic enhancer fertilization process is completed. The intergenic enhancer interacts with the FTO promoter and its intronic enhancer during this phase, resulting in FTO activation during the preimplantation stage. As a result, the progeny will be actively transferred in the continuance of gene expression [71,77].

On the other hand, BPA is able to modify and increase the expression of mRNA in the DNA methyltransferases, histone methyltransferases, and their downstream proteins in the neonatal germ cells on prenatal exposure with BPA [78]. This epigenome alteration is further supported by the studies done by Alonso-Magdalena et al. (2016) that show that inheritance of *TNFRSF12A*, *ESRRA*, *FGF19*, *WNT10B*, *GDNF*, *ENOPH1*, *ATF3*, *NCAM2*, *NTF3*, *PITX3*, and *DPYSL2* as evidence of obesity development in the offspring [79]. Another commonly observed epigenetic pattern is the alteration in IGF2 methylation in the male germline, which results in β-cell dysfunction and impaired glucose tolerance in the offspring [80]. Impaired glucose tolerance enhances the downregulation of Pdx1, causing the miR-338 to be upregulated. This will be eventually passed down to the offspring and Chang et al. (2016) observe a suppression in Pdx1 expression, causing deacetylation of histone type 3 and 4 in the F1 generation. This process happens through the methylation action of H3K9 and the demethylation action of H3K4 [81]. Since glucose metabolism plays a vital role in spermatogenesis, impaired glucose tolerance greatly affects the normal function and structure of the sperm. In female subjects, stromal cell loss due to the increased level of oxidative stress, and the decreased level of SOD, GSH, or GPx may further enhance the transmission of the defects through reproductive organs [16].

Another study shows that BPA exposure in prenatal interferes with miRNA expression, causing 15 related genes to insulin signaling to be affected. As a result, resistance towards insulin rises, leading to reduced hepatic glycogen storage in the F1 generation. Simultaneously, hepatic DNA methylation will be reduced due to the activation and overexpression of DNA methyltransferase 3B mRNA. As such, the synthesizing of glycogen will be hindered as a cause of hypermethylation of hepatic glucokinase [83]. Junge et al. (2018) notice prenatal BPA exposure alters methylation at CpG sites at the mesoderm-specific transcript gene. This mesoderm-specific transcript gene encodes for α/β hydrolase an obesity-associated gene in the paternal and possesses the isoform imprinting that can be transmitted to the offspring [84].

### 3.7. BPA and Cardiovascular Complications

Obesity develops when NPY and AgRP levels rise as a result of prolonged or frequent BPA exposure. In response to this assertion, in the event of BPA exposure, the activation of the same neurons contributes to cardiac disease. As one of the key regulators of the heart, NPY can directly interact with cardiac nerves. NPY, on the other hand, has the capacity to act as both a cardiac stressor and a depressant. This effect is primarily influenced by the ligand concentration, receptor expression, and the presence of adrenergic and DPP4 activity in the heart. Alike in obesity, the pathogenesis of hypertrophic cardiomyopathies, hypertension, myocardial ischemia, heart failure, and atherosclerosis is closely associated with Y1 and Y2 receptors. NPY affects protein degradation, cardiac contraction, and cardiomyocyte proliferation via the autocrine or paracrine mechanism [85]. In contrast, Y2 receptors are responsible for the enhancement of Ito postsynaptic stimulus, inhibition of presynaptic neurotransmitter release and reduction of Ito in cardiomyocytes [86]. BPA could trigger the expression of NPY via the sympathetic nerve. Because an increase in NPY merely reflects an increase in the density of sympathetic nerve terminals. Sympathetic nerves link to the parasympathetic nerves via the Y2 receptor on the surface of parasympathetic nerves. As a result, the release of acetylcholine is suppressed, preventing the heart from relaxing, resulting in bradycardia and coronary artery spasm. Overexpression of NPY stimulates angiogenesis in blood vessels by stimulating the release of VEGF and enhancing the process of mitogenesis or vasoconstriction in endothelial cells, resulting in decreased coronary blood flow and cardia output [85], endothelial dysfunction, increased peripheral resistance, and the formation of local vascular stenosis. On the other hand, the release of VEGF does contribute to plaque rupture and hemorrhage [87]. Table 2 summarizes the effect of BPA on cardiovascular disease.

On the contrary, NPY possesses the characteristics to stop the effect of anti-angiogenic agents in cardiac such as endostatin and angiostatin [88] thereby preventing the revascularization of ischemic tissue [86] and the thickening of the intimal tissue in the heart [87]. Furthermore, NPY increases the release of dipeptidyl peptidase 4 in heart tissue under specific clinical conditions. As a result, there is an imbalance of interaction in sympatho-vagal, cardiac contraction, and remodeling, which leads to endothelium dysfunction, uncontrolled angiogenesis, and proliferation in cardiac tissue. As a result, the risk of cardiac arrhythmias, hypertrophy, ischemia, and even heart failure increases [85]. According to studies, NPY inhibits the activity of adenylate cyclase, N-type Ca^2+^, and nicotinic cholinergic currents in the heart, and triggers the synthesis of inositol phosphates and the activation or inhibition of inwardly rectifying K^+^ [89]. NPY may also activate the MAPK/ERK, PI3K/Akt, and G_q_ molecular pathways, causing Ca^2+^ to be released from the sarcoplasmic reticulum via inositol triphosphate. Ca^2+^/calmodulin-dependent kinase is activated as a result of the increase in intracellular Ca^2+^. This is followed by increased PKC activation via diacylglycerol [85], which leads to cardiac hypertrophy or failure [90]. From the context of AgRP, AgRP increases hunger by acting directly on the CNS, hence weight gain is a typical clinical manifestation. According to studies, this results in a persistent drop in mean arterial pressure and heart rate. Although the underlying molecular mechanism is unknown, scientists believe it is connected to MC3/4R, an AgRP antagonist found in the hypothalamus. It is speculated that the antagonism action of AgRP could potentially inhibit the sympathetic activity in adipose tissue in addition to the stimulation of pressor and tachycardiac in heart due to chronic central MC3/4R activation [91].

**Table 2:** BPA and cardiovascular disease.

Author	Subjects	Type of Exposure	Duration of Exposure	Dosage	Findings
Apaydin et al., 2019 [92]	Adult male albino rats	Oral gavage	28 days	130 mg/kg/bw/day	- Increased level of malondialdehyde (MDA) - Decreased level of catalase, SOD, Glutathione-S-transferases (GST), and glutathione peroxidase (GPx). - Presence of congestion, muscle degeneration, necrosis cytoplasmic edema, vacuolization in mitochondria, dilation in the sarcoplasmic reticulum, and inflammatory infiltration were seen in myocardial fibers.
Bruno et al., 2019 [93]	5 week old adult female balb/c mice	Drinking water	2 weeks	2.5, 25, and 250 µg/L	- Increased level of inflammatory infiltration in foci. - Increased level of viral myocarditis and pericarditis. - Increased level of CD4^+^ T cells, mast cells, and degranulation of mast cells in heart tissue. - Increased expression of ERα, ERβ, IFNγ and IL-17A cardiac tissue. - Increased level of TLR4, Caspase-1, and IL-1β in cardiac tissue. - Presence of fibrosis was seen in pericardium, vessels, and myocardium.
Reventun et al., 2020 [94]	8 weeks old male wild-type CD1 mice	Drinking water	16 weeks	4 × 10^−7^ M	- Impaired cardiac contraction and heart enlargement is seen. - Increased thickness of diastolic and systolic interventricular septum. - Increased level of collagen type I expression and cardiac ischemic is observed. - Decreased cardiac output and ventricular diameter is seen. - Increased level of macrophage infiltration, CD68^+^, TNF-α, chemokine, TFG-β, IL-10, and ligand 7 in the cardiac tissue. - Presence of edema in the cardiac interstitial tissue, hemorrhagic lesion, and injured vessels within the heart was seen. - Increased level of permeability in vessels and necroptosis in endothelial cells was observed.
Bahey et al., 2019 [95]	Adult male Wistar rats	Intraperitoneal injection	3 weeks	1.2 mg/kg/day	- Increased intracellular space in myocardial fibers. - Disorganization of muscle fibers in heart. - Congestion in blood vessels, extravasation of red blood cells in heart, and absence of nuclei in cytoplasm is seen. - Mononuclear cell infiltration and increased level of cardiomyocytes, and fibrous tissue were seen in cardiac tissue. - Dilation of intramyocardial in blood vessels was observed.
Brown et al., 2019 [96]	3rd generation homozygous TG(ERE:GFP)Casper *Danio rerio* zebrafish	Water in a static system	6 h post fertilization to 5 days of post fertilization	25 and 1000 μg/L	- Decreased level of collagen filaments, dislocation of valvular cells, and misshapen in the leaflets of heart valves is seen. - Downregulation of *esr1* and *esr2* signaling.
Friques et al., 2020 [97]	3 weeks old male Wistar rats	Oral gavage	60 days	100 μg/kg/day	- Increase in systolic and diastolic blood pressure. - Decreased Ach induced relaxation activity in the aortic rings. - Decreased vasodilator response towards Ach and endothelial dysfunction in cardiac muscles. - Increased level of O_2_^−^, H_2_O_2_, and DNA fragmentation in aorta cells. - Necrosis of aorta cells, irregularity, and increased spaces in endothelial cells were observed.
Amin, 2019 [98]	Adult Wistar rats	Subcutaneous injection	6 days	30 mg/kg/day	- Increased spaces, sarcoplasmic vacuolation, and TLR2 were seen in the cardiomyocytes. - Congestion and dilation in blood vessels, extravasation of red blood cells in heart were seen. - Irregular scattered nucleus with thickened heterochromatin, swollen mitochondria, and distorted intercalated disc were seen in the cardiomyocytes.
Makowska et al., 2021 [99]	8 weeks old female juvenile pigs mixture of Piétrain and Duroc breed	Capsules	28 days	0.05 mg/kg/day	- Increased level of NPY and immunoreactive nerves for tyrosine hydroxylase, and vesicular acetylcholine transporter in the apex of the heart.
Lombó et al., 2019 [100]	4 months old wild type *Danio rerio* zebrafish	Water in a static system	24 h post fertilization	2000 and 4000 μg/L	- Development of edema, ballooning, defect in the loops, elongated heart chambers, and accumulation of blood were seen within the heart. - Increased expression of heart and neural crest derivatives expressed 2 (*hand2*) gene. - Increased level of lysine 9, 12, and 14 acetylation.
Valokola et al., 2018 [101]	Adult male Wistar rats	Oral gavage	4 weeks	10, 25, and 50 mg/kg	- Increased level of MDA, systolic, diastolic pressure. - Decreased level of GSH in the heart. - Elevation of QT and PQ on the ECG. - Inflammatory infiltration in the heart muscles. - Enlarged nucleus with increased of hollow spaces in the cytoplasm of myocytes was seen.
Rameshrad et al., 2018 [102]	Male albino Wistar rats	Oral gavage	2 months	35 mg/kg/day	- Increased level of MDA in the aorta tissue. - Decreased of the vasoconstriction response of aorta rings. - Increased level of vascular cell adhesion molecule, and cleaved caspase 3 protein.
Eweda et al., 2019 [103]	Adult male Wister albino rats	Oral	6 weeks	30 mg/kg	- Increased level of AST, ALT, and bilirubin in the liver. - -Increased level of TG, total cholesterol (TC), LDL and VLDL. - Decreased level of HDL, SOD, GSH, GPx in the liver. - Accumulation of lipids in the liver is seen. - Increased level of CK-MB and LDH in the liver. - Degeneration, hemorrhagic lesion, aggregation of lymphocytes, and dilation of sinusoids were observed in the hepatocytes. - Loss of sarcoplasm fragmentation, striations, architecture, presence of edema, congested blood vessels, vacuolization in the cytoplasm, and thickening of the coronary branches were visible within the cardiac muscles. - Atrophy of elastic fibers, loss of endothelial cells, and changes in the sclerotic wall were seen within the tunica media.
Khodayar et al., 2018 [104]	6 weeks old male Wistar rats	Oral	30 days	50 mg/kg	- Increased level of MDA, AST, LDH, and CK-MB in the liver. - Increased level of TG, TC, and LDL. - Decreased level of SOD, CAT, GSH, and GPx in the heart. - Loss of myofibrillar tissue, red blood cell congestion, disorganization of myocytes, and inflammatory infiltration in the heart.
Sivashanmugam et al., 2017 [105]	12 to 14 weeks old male albino Wistar rats	Oral	30 days	10,100, and 400 mg BPA/kg	- Increase of fasting blood glucose. - Decreased level of insulin receptors and pIR^Tyr1162^ protein. - Decreased level of Akt, and pAkt^Ser473^ protein phosphorylation. - Decreased level of GLUT4 and cardiac muscle fraction in the plasma membrane.
Kasneci et al., 2017 [106]	C57bl/6n mice	Drinking water	22 days	25 ng/ml	- Increase of body weight and cardiac hypertrophy were seen. - Cardiac rupture and increased dilation of systolic and diastolic were observed. - Increase of dilation of left ventricle and cardiac contraction. - Increase of monocytes and myeloid cell infiltration in the heart. - Accumulation of CD4^+^ T cells and CD8^+^ in cardiac tissue. - Increased expression of metalloproteinase-1 protein, ARG1, IL-4, NOS2, and COX2 in heart.
Prudencio et al., 2021 [107]	3 to 4 months old female Sprague Dawley rats	Not specified	Not specified	0.0–100 µM	- Inhibition of calcium channels (type T and L). - Calcium leakage from sarcoplasmic reticulum was seen. - Reduced level in FPD, heart rate, and atrioventricular conduction. - Increase of atrioventricular nodal refractory.
Oluranti et al., 2021 [108]	Wistar rats	Oral	28 days	25, and 50 mg/kg	- Decreased level of SOD, CAT, GSH, and Nrf2 in the myocardial tissue. - Increased level of MDA, CRP, and NF-κB in the myocardial tissue.
Rasdi et al., 2020 [109]	6 to 8 weeks old female Sprague Dawley rats	Drinking water	Pregnancy day 2 up to 21 days	0.05 and 0.2 mg/ml	- Presence of fibrosis and muscle remnants in the cardiac tissue. - Increase of systolic and diastolic blood pressure. - Cardiac hypertrophy and injuries to cardiac muscles were seen. - Expression of cardiac troponin I was observed in the fetus heart.
Gear et al., 2017 [110]	Female Sprague Dawley rats	Oral gavage	6 months	2.5, 25, 250, 2500, and 25,000 μg/kg/day	- Increase of heart weight. - Accumulation of collagen IV within the heart. - Increased thickness in the left ventricles, presence of fibrosis, and inflammatory infiltration were seen in the heart. - Degeneration, vacuolation, disorganization, and fibrosis were seen in the myocytes. - Macrophage infiltration and degeneration were seen in the myofibrils.
Vanani et al., 2020 [111]	8 to 10 weeks old male Wistar rats	Intragastric intubation	14 days	250 mg/kg	- Increased level of CK-MB, TG, LDL-C, and LDH in serum. - Decreased level of HDL in the serum. - Increased level of ROS, MDA, and membrane potential in the heart’s mitochondria. - Decreased level of GSH and CAT in the mitochondria.

## 4. BPA Interaction with Specific Receptors in Obesity

Generally, the binding of BPA to estrogen receptors (ERα and ERβ) in obesity is well known. Recent investigations prove that BPA binds with estrogen receptors with a force of 42 van der Waals, leading to the activation of different pathways which contribute to obesity. The similar features in adipogenic transcription and interaction of BPA with estrogen receptors further ease the development of obesity [112]. However, recent studies have disclosed different receptors such as androgen receptor, GPR30, glucocorticoid receptor, and estrogen-related receptor gamma (ERRγ) have been hypothesized linked to obesity in association with BPA action.

### 4.1. Androgen Receptor

BPA has the potential to bind with androgen receptors which can suppress the expression of androgen. In other words, BPA disrupts the activation of androgen and exhibits anti-androgenic effects [113]. The secretion of testosterone, a most common form of androgen determines the pattern of fat distribution in a sex-dependent manner [114]. The reduced level of testosterone in males increases central adiposity. Basically, adipocytes contain a very concentrated amount of aromatase. Aromatase is an estrogen synthetase that transforms testosterone into oestradiol. Thus, free androgen levels in the circulatory system will be increased. Simultaneously, oestrogen will suppress the luteinizing hormone and gonadotropin-releasing hormone via the action of hypothalamo-pituitary axis. Resultantly, the production of testosterone will be further reduced. This low level of testosterones enhances adiposity through negative feedback of hypogonadal obesity cycle in males [115].

### 4.2. GPR30

BPA’s lipophilic features ease the binding of BPA to *GPR30* and stimulate the overexpression of *GPR30* in fat cells. This is because *GPR30* is known as a membrane protein which has the ability to bind with estrogen and activates its intracellular signaling pathway [116]. In such a case, the adiponectin levels will be lowered, preventing lipolysis as discussed earlier. However, the activation of *GPR30* could be due to the increased expression of FAS and phosphorylation of ERK1/2 in mature adipocytes. This will eventually activate its downstream signaling molecules such as *GPR30*, in mature adipocytes [117], thereby increasing the rate of proliferation in the adipocyte cells and secretion of cytokines by adipocytes [118]. As a result, the mass of the fat cell will increase, subsequently contributing to obesity over time [119].

### 4.3. Glucocorticoid Receptor

BPA is able to bind with glucocorticoid receptors and exhibit against action. As such, upon binding the downstream molecules of glucocorticoids such as CCAAT/enhancer-binding protein β (C/EBPβ) will be stimulated followed by C/EBPα and PPARγ transcription factor pathway. Since C/EBPβ plays a pivotal role in preadipocyte differentiation, the activation of C/EBPβ will eventually stimulate adipogenesis and lead to over-accumulation of fats [120].

### 4.4. Estrogen-Related Receptor Gamma (ERRγ)

Study shows that BPA could bind with *ERRγ* with a high affinity due to the presence of a crystalized shaped ligand-binding domain in the *ERRγ.* A nanomolar concentration of as low as 5.6 BPA is enough to bind and stimulate the transcriptional activity of *ERRγ*. *ERRγ* is responsible to modulate the expression of *Pck1*, a glucogenesis gene [121]. In this context, a knock-out of *Pck1* will result in suppressed glyceroneogenesis activity and decreased level of re-esterification process in fatty acid. As a consequence of this, lipodystrophy emerges [122]. This might be the primary underlying reason for ERRγ as the major element in contributing to infants’ obesity [121].

## 5. BPA Exposure and Adverse Perinatal, Childhood, and Adult Cardiovascular Health Outcomes

There is a growing body of evidence indicating a relationship between early exposure to BPA with the development and progression of cardiovascular disease throughout a human’s life. Prolong exposure of adults to BPA has been associated with cardiovascular and hypertension disease. Higher urinary BPA was significantly found in patients diagnosed with cardiovascular disease like angina, coronary heart attack and heart attack. Higher BPA concentrations (>0.00048 μg/mL) are positively associated with coronary heart disease [123]. BPA has been shown to have long term effects. A 10-year prospective study in the United Kingdom done by Melzer et al. (2010) found higher BPA exposure, reflected in higher urinary concentrations of BPA, is consistently associated with a higher incidence of coronary heart disease [124]. Besides that, high levels of urine BPA are also shown to be linked to a higher prevalence of peripheral artery disease in adults. Hypertension is well known as a significant risk factor for CV diseases. Studies have proven that individuals diagnosed with hypertension are found to have increased total urinary BPA. BPA exposure also can cause a decrease in heart rate variability (HRV). Decreased HRV together with increased blood pressure is reported to increase the chance of developing cardiovascular disease [125,126]. A recent study done by Kataria et al. (2017) reveals that BPA exposure can induce an oxidant stress-induced injury to the endothelium and may lead to its dysfunction [127].

A study done by Kubo et al. (2004) proposes that BPA promoted HIF-1alpha (hypoxia-inducible factor) degradation in the presence of cobalt and prevents the induction of erythropoietin which responds to hypoxia. Disturbance in the development of the respiratory system could cause infants to experience intermittent hypoxia [128]. Hypoxia is believed to be responsible for the occurrence of cardiovascular diseases like stroke and myocardial infarction. Other than that, it is also suggested that BPA exposure during pregnancy may alter the fetal epigenome, altering the shape and function of the fetal heart, potentially leading to a variety of cardiovascular diseases in children and adults [123]. The placenta has a huge and important role in fetal development and growth. It is considered a chief regulator of nutrient and oxygen supply to the growing embryo during gestation. Any disturbance or changes in the structural and functional of the placenta may cause adverse health outcomes to the neonate. BPA has been associated with the changes at the molecular level, especially in the placental micro-RNA expression, DNA methylation, and genomic imprinting [129].

## 6. Biomarkers

The detection of biomarkers is essential in the clinical setting to prevent any unwanted adverse effects and biomarkers are an excellent diagnostic tool to rule out the causative element and the underlying mechanism of a particular condition. Physicians usually focus on changes in hormone levels such as testosterone, follicle-stimulating hormone, and cortisol to access the enzymatic activity in subjects exposed to BPA. This is because changes in such hormones are indicative of homeostatic interruption. For instance, extreme levels of cortisol are indicative of interference of BPA in the hypothalamic–pituitary–adrenal axis, while AST, ALT, and lactate dehydrogenase are indicators for liver damage in an attempt to remove BPA metabolites [130]. Different biomarkers than the abovementioned biomarkers serve as an ideal diagnostic tool if the subjects refer to hospitals with any underlying conditions such as subjects exposed with BPA with ovarian cancer; the presence of *keratin 4* genes is vital to confirm the diagnosis [130]. However, visfatin, resistin, leptin, or haptoglobin are ideal biomarkers for adiposity. Specifically for BPA-associated obesity, C-reactive protein, Von Willebrand factor, or sialic acids are good predictors and biochemical parameters that are necessary for clinical diagnostics [131].

Apart from this, there are few biomarkers that indicate BPA exposure in humans. In regard to this statement, sugar metabolism is considered to be the most effective procedure in determining the exogenous BPA exposure while metabolomics is known for its sensitiveness in determining health hazards risk due to BPA. For instance, from a metabolomics perspective, excessive levels of lactate and choline have been proven to be closely linked to BPA exposure. The presence of lactate and choline can be detected even less than 1 mg/L or 25µg/kg/day of BPA exposure. This applies to prenatal BPA exposures too [132]. Lactate and choline can be measured via blood plasma in normal subjects while in fetuses, it can be detected through amniotic fluid or scalp [133]. Urine is another method of biomarker detection for BPA exposure. In urine, different forms of BPA can be measured. This includes unconjugated BPA, BPA glucuronide, total BPA, or even BPA sulfates. This method is considered to be ideal since BPA is a hydrophilic chemical with semivolatile properties, urine contains the highest concentration of metabolites with a minimal risk of external contamination, and the BPA content can be detected in the range of ng/mL [134].

Other than this, 8OHdG, 8-isoprostane, and MDA can be spotted in the urine that results from oxidative stress due to BPA [135]. The presence of 8OHdG, a marker for oxidative stress, induces DNA damage while 8-isoprostane is an indicator for lipid peroxidation due to the BPA toxicokinetics mechanism [136]. The transgenerational effects biomarker for BPA can be detected via the expression of the Kisspeptin gene in the placenta. Since BPA is also known for its reprotoxicant characteristics, it could affect the hypothalamic pituitary gonadal axis, and lead to obvious transgenerational effects [135]. DNA methylation of BDNF in region IV, a neurotrophin that is present in the brain which affects neurocognitive developments can be spotted in blood. Studies show that downregulation of BDNF in offspring is one of the notable clinical signs observed in prenatal fetuses exposed to BPA [137]. 3-nitrotyrosine is another common biomarker that can be noticed in BPA-exposed subjects. 3-nitrotyrosine is said to be raised in the plasma of pregnant mothers and umbilical cord due to nitrosative stress. Similar effects were witnessed in rodents and pregnant sheep exposed to BPA [138].

## 7. Safety Concerns and Dosage

In consideration of BPA side effects, some nations, such as Canada, France, and the European Union, have outright prohibited the use of BPA in favor of bisphenol S and bisphenol F [139]. According to the FDA in 2014, a dosage of 50 mcg/kg/day, which is equivalent to 23 mcg/pound/bw, is considered safe. However, animals exposed to a dose similar to or lower than this (10 mcg/kg) had adverse effects [140]. According to the toxicological research report, the active form of BPA is undetectable in fetuses up to 8 h [141]. In toto, the daily consumption of BPA is deemed to be tolerated at 10% of total body weight, or 0.05 mg/kg [142].

## 8. Conclusions

In conclusion, this review summarizes the underlying mechanism of BPA in obesity. Exposure to BPA enhance adipogenesis, lipid dysregulation, and adipose tissue inflammation, thereby increasing the risk of obesity and the presence of a high concentration of BPA in serum and urinary are the evidence for this. The articles included in the reviews specify that BPA could induce transgenerational effects in obesity and can last up to F5 generation. Due to similar pathway involvement in BPA, cardiovascular complications in obesity are also one of the most notable signs. However, the effect can be sex-dependent. The usage of BPA is impossible to be banned completely. However, it is highly recommended to use alternatives such as BPS.

## 9. Future Directions

The literature search in this study is limited to animals and the evidence of BPA exposure and obesity is still something in which the conclusions concerning human health effects are debatable. There is no definite safe dosage that has been suggested for humans except on the basis of animal study evidence. As such, the association between BPA and obesity in humans is still lacking. There is a high possibility that the conclusion drawn from animal studies could not be valid to be practiced for humans. As such, there must be more studies focusing on BPA exposure in humans that must be done in the future. In this way, the undiscovered underlying mechanism can be studied and the issue of the global obesity epidemic can be prevented by the modification of the genes involved. Perhaps in the future, we can focus on the effects of BPA in each stage of the fetus development up to birth in association with obesity. This research gap will surely give insights to researchers to develop a more reliable intervention for obesity in the future. In due course, there is a clear need for studies focusing on pre-intervention to reverse obesity. Future research should focus on the alternative method for BPA usage or limit the leakage of BPA from manufactured products. In prioritizing global health, policies should be constructed to prohibit the usage of BPA in food containers, particularly in baby feeding bottles.

## Figures and Tables

**Table 1:** BPA and obesity: in vivo study.

Author	Subjects	Type of Exposure	Duration of Exposure	Dosage	Findings
Desai et al., 2018 [14]	12 weeks old female Sprague–Dawley rats	Oral (purified drinking water containing BPA)	Before 2 weeks of mating up to weaning stage	5 mg/L/day	- Gradual increase of BPA in maternal plasma. - Increase in offspring body weight. - Increase in systolic blood pressure of the offspring. - Increased level of adipogenic transcription factor PPARγ expressionin offspring. - Increase mass of adipose tissue and hypertrophic adipocytes in male offspring. - Increased level CD68 and TNF-α in adipose tissue of the offspring.
Shih et al., 2021 [15]	15 weeks old female Sprague–Dawley rats	Oral gavage	6th day after pregnancy up to 36 days	50 µg/kg/day	- Increased level of abdominal lipid weight up to 77% in female offspring. - Decreased level of HDL up to 49%. - Increased level of TG, TC, LDL, and leptin. - Increased level of *Prevotella*, *Clostridium perfringens*, and *Clostridium ruminantiums* in feaces. - Increased level of acetate concentration in feaces in female offspring.
Dabeer et al., 2019 [16]	3 to 4 weeks old male and female Wistar rats	BPA in drinking water	180 days before mating and up to postnatal day (PND) 35	10 ppm and 10 mg/L/day	- Gradual increase in weight of the female offspring. - Increased level of abnormal sperm in male offspring. - Increased level of fasting plasma glucose in both male and female offspring. - Increased level of plasma cholesterol, TG, and lipid peroxidation in male offspring. - Decreased level of HDL and superoxide dismutase (SOD) in male offspring.
Taylor et al., 2018 [17]	3 month old nulliparous female CD-1 mice	Oral gavage	Pregnancy confirmation up to PND 22	5 or 500 mg/kg/bw/day	- Increased body weight and gonadal fat of the prenatal mice. - Hypermethylation of *gm4788*, *rrp15*, *gm4222*, *ctnnbl1*, *skint10*, *zfp420*, *cep290*, *d16ertd472e*, *pde10a* genes. - DNA demethylation at the transcription start site. - Strong expression of *fggy* mRNA.
Santangeli et al., 2018 [18]	Wild type adult female *Danio rerio*	Water in a static system	21 days	5 μg/L, 10 μg/L, and 20 μg/L	- Increased level of hepatosomatic index. - Increased level of SREBP-1, acyl-coenzime A cholesterol acyltransferase (ACAT2), CCAAT/enhancer binding protein α (CEBPA), fat storage-inducing transmembrane protein2 (FITM2), and fatty acid binding protein 1A (FABP11A). - Decreased level of diglyceride acyltransferase (DGAT2) and PPARα. - Increased level of lipid concentration in liver.
Malaisé et al., 2017 [19]	8 week old female and male C3H/HeN mice	oral	PND 170	50 µg/kg/day	- Increase in body weight in the offspring. - Increased level of glucose tolerance in the offspring. - Decreased level of insulin sensitivity gradually in the offspring. - Increased level of IL-17, IL-22, TNF-α and IFN-γ in the liver. - Decreased level of IgA and pIgR formation. - Decreased level of *Bifdobacterium* spp. in the feaces.
Pu et al., 2017 [20]	Primiparous female sheep	Subcutaneous injection	147 days	0.5 mg/kg/day	- Increased level of PPARγ mRNA expression in fetal adipose tissue. - Increased level of differentiation rate in adipocytes. - Increased gene expression of *GR*, *ESR1*, *ESR2*, and *ERRα*. - Increased expression of *FABP4, GLUT4*, and *SOX6* in the offspring. - Increased expression of unfolded protein response in the offspring.
Stoker et al., 2019 [21]	90 days old female Wistar rats	BPA in drinking water	Gestation day 9 to weaning	50 µg/kg/day	- Increased fat composition in epididymal and perirenal in the offspring. - Stimulation of hyperphagia in the offspring. - Increased level of fasting serum glucose and leptin in the male offspring. - Orexigenic neuropeptide expression is seen in the hypothalamus of the male offspring.
Neier et al., 2019 [22]	12 to 27 weeks old C57BL/6J female mice	Chow diet	Gestation day 9 to PND 21	50 μg/kg/day	- Gradual increase of body weight in the offspring. - Increased level of fat in the gonadal, messentric, and subcutaneous tissue of the offspring. - Increased level of oxidizing glutathione in the offspring.
Tian et al., 2021 [23]	5 months old wild type adult male *Danio rerio*	Water in a static system	28 days	20, 100, and 500 μg/L/day	- Increase in body weight, length, and food intake in male subjects. - Accumulation of lipid in the liver of the larvae. - Increased level of leucine, isoleucine, valine, and acetate in the larvae and adult zebrafish. - Decreased level of inosine in the larvae and adult zebrafish. - Severe fatty changes in the microvesicular, ballooning of the hepatocyte, infiltration of inflammatory cell, and pyknotic nucle is seen. - Upregulation of insulin signaling pathways, and cannabinoid receptor type (*CB1*). - Decreased level of *gpr55*, fatty acid amide hydrolase and expression of PPARα in the liver and adipose tissue.
Lin et al., 2019 [24]	3 weeks old male Wistar rats	Drinking water	8 weeks	1 µg/mL/day	- Increased deposition of fat in the visceral and liver. - Increased level of total cholesterol, TG, LDL-C, IL-17, and TNF-α in the plasma. - Decreased level of HDL in the plasma. - Condensed hepatocytes, diffused cytoplasm, and lipid droplets were seen in the liver tissue. - Upregulation of SREBP1 and ACC1 mRNA gene. - Decreased level of HSL, ERα and ZAG protein in the liver. - Increased level of TLR4 and NF-κB protein in the liver.

## Data Availability

Not applicable.
